# Central nervous system involvement and thrombocytopenia as predictors of mortality in children with hemophagocytic lymphohistiocytosis

**DOI:** 10.3389/fped.2022.941318

**Published:** 2022-09-06

**Authors:** Saralee Harnchoowong, Sirisucha Soponkanaporn, Soamarat Vilaiyuk, Butsabong Lerkvaleekul, Samart Pakakasama

**Affiliations:** ^1^Division of Rheumatology, Department of Pediatrics, Faculty of Medicine Ramathibodi Hospital, Mahidol University, Bangkok, Thailand; ^2^Division of Hematology and Oncology, Department of Pediatrics, Faculty of Medicine Ramathibodi Hospital, Mahidol University, Bangkok, Thailand

**Keywords:** hemophagocytic lymphohistiocytosis (HLH), macrophage activation syndrome (MAS), mortality rate, prognostic factor, pediatric patients

## Abstract

**Introduction:**

Hemophagocytic lymphohistiocytosis (HLH) is a potentially life-threatening condition. This study aimed to evaluate treatment outcomes and identify prognostic-related factors in Thai children with HLH.

**Materials and methods:**

We retrospectively reviewed the medical records of 76 pediatric patients with HLH who were treated at Ramathibodi Hospital between January 2004 and December 2019. Treatment outcomes were defined as early mortality (death within 30 days after diagnosis) and early treatment response (resolution of all clinical features and normalization of at least one HLH-related laboratory parameter within 4 weeks).

**Results:**

The overall mortality rate was 38% (29/76), with an early mortality rate of 45% (13/29). Malignancy-associated HLH had the highest mortality rate (88%), followed by primary HLH (56%). The predictors of early mortality were central nervous system (CNS) involvement [OR 13 (95%CI 2–83), *p* = 0.007] and platelet counts <44 × 10^6^/mm^3^ [OR 8 (95%CI 1.3–49), *p* = 0.024]. The predictors of early treatment response were no CNS involvement [OR 6.6 (95%CI 1.5–28.8), *p* = 0.011], platelet counts more than 44 × 10^6^/mm^3^ [OR 8 (95%CI 2.1–30.9), *p* = 0.003], and total bilirubin levels <1.8 mg/dL [OR 4 (95%CI 1.1–14.8), *p* = 0.036]. In the mixed-model analysis, platelet counts in non-survivors increased significantly less than those in survivors, with a mean difference in platelet changes between the two groups of 94.6 × 10^6^/mm^3^ (*p* = 0.003).

**Conclusion:**

The independent predictors of early mortality in children with HLH were CNS involvement and low baseline platelet counts. A slow rate of platelet increases during the first week after diagnosis was also associated with mortality.

## Introduction

Hemophagocytic lymphohistiocytosis (HLH) is a rare life-threatening condition characterized by persistent activation of T lymphocytes and macrophages, leading to the hypersecretion of pro-inflammatory cytokines, including interleukin (IL)-1, IL-6, IL-18, tumor necrosis factor (TNF), and interferon-gamma (IFNγ) (1). HLH is classified as primary and secondary HLH. Primary HLH is caused by genetic mutations affecting granule-dependent natural killer cell cytotoxicity and cytotoxic T lymphocytes. The main subtype of primary HLH is familial HLH (fHLH), which had specific gene mutations. Known genotypes that have been reported in fHLH are *PRF1, UNC13D, STX11*, and *STXBP2* genes (2–5). The other primary HLH subtype is related to primary immune deficiencies with defects in vesicle or lysosome synthesis, trafficking, and release, leading to T cell, macrophage, and neutrophil dysfunctions (6). Primary HLH occurs most commonly in patients with younger age, a family history of HLH, or recurrent episodes (7). Secondary HLH is an acquired condition that may be triggered by infections (infection-associated hemophagocytic syndrome or IAHS), rheumatic diseases (macrophage activation syndrome or MAS), and malignancies (malignancy-associated HLH or M-HLH). The common underlying rheumatic diseases in children are systemic juvenile idiopathic arthritis (SJIA), systemic lupus erythematosus (SLE), and Kawasaki disease (8–11). However, recent studies showed that some patients with secondary HLH contained heterozygous fHLH-related mutations which associated with poor outcome (12, 13).

The treatment of HLH varies across subtypes, and outcomes often depend on how early patients are diagnosed. It is challenging to differentiate HLH from sepsis and active underlying disease because the symptoms are quite similar. In the late stage of HLH, patients rapidly deteriorate and have multiple organ dysfunction, causing high morbidity and mortality. The overall mortality rate in children with HLH is 20–40%, with higher mortality in specific subtypes (50% in primary HLH and 56% in M-HLH) and lower mortality rates in patients with MAS (8%) (1, 8, 14). Early identification of high-risk patients is essential for improving treatment outcomes. Previous studies identified risk factors of poor treatment outcomes in children with HLH and found that central nervous system (CNS) involvement was associated with a lower survival rate (15). A study of non-malignancy-associated secondary HLH showed that factors related to worse prognosis included low levels of hemoglobin, platelets, and albumin (16). Prolonged partial thromboplastin time (PTT), high levels of lactate dehydrogenase, and increased ferritin also predicted mortality in children with HLH (17–19).

In addition to initial laboratory findings, sequential changes in laboratory parameters can help predict treatment outcomes. However, only a few studies have investigated changes in laboratory markers. Lin et al. found that a reduction in ferritin by <50% was associated with higher mortality than ferritin decreases by more than 96% (20). In an adult study, patients with a reduction in ferritin by more than 35% from the peak level had a higher chance of survival, whereas patients with an increase in ferritin by more than 35% were likely to die (21). In addition, a study in children with HLH found that platelet normalization within 2 weeks was a predictor of disease resolution (22). However, no study has investigated the association between the magnitude of change in hematologic laboratory parameters and treatment outcomes. Here, we aimed to evaluate disease outcomes and identify predictive factors for treatment outcomes and mortality risk in Thai children with HLH.

## Materials and methods

### Study design and patients

This was a retrospective cohort study. Patients under 18 years diagnosed with HLH between January 2004 and December 2019 at Ramathibodi Hospital were included. The diagnosis of HLH was based on HLH-2004 criteria, which required molecular genetic diagnostic confirmation or fulfillment of at least five out of the following criteria: (1) fever, (2) splenomegaly, (3) cytopenia for at least two lineages (hemoglobin level <9 g/dL, neutrophil count <1,000/mm^3^, or platelet count <100 × 10^6^/mm^3^), (4) triglyceride level more than 265 mg/dL or fibrinogen level <150 mg/dL, (5) evidence of hemophagocytosis in bone marrow, spleen, or lymph nodes, (6) low or absent natural killer cell activity, (7) ferritin level more than 500 ng/mL, and (8) soluble CD25 level more than 2,400 U/mL (23). Because assessments for natural killer cell activity and soluble CD25 levels were not available, we included patients with at least five out of the other six criteria. Patients with primary HLH were diagnosed by positive molecular genetic confirmation, a family history of HLH, or young age of onset with recurrent episodes. The diagnosis of MAS in SJIA patients was based on the 2016 classification criteria of MAS in SJIA, including febrile patients with ferritin levels more than 684 ng/mL and any of two of the following: (1) platelet count <181 × 10^6^/mm^3^, (2) aspartate aminotransferase (AST) level more than 48 U/L, (3) triglyceride level more than 156 mg/dL, and (4) fibrinogen level <360 mg/dL (24). Patients were diagnosed with SJIA according to the International League of Associations for Rheumatology classification (25). This study was approved by the Ethics Committee of the Faculty of Medicine, Ramathibodi Hospital, Mahidol University, Thailand (MURA2020/393) and conducted following the principles of the Declaration of Helsinki.

### Data collection

The medical records were retrospectively reviewed. Demographic data and baseline clinical characteristics, including age, gender, underlying disease, duration of disease, initial manifestations, and treatment (medications and platelet transfusions during 1 week after diagnosis), were obtained. CNS involvement was indicated when patients had symptoms of seizure, irritability, altered consciousness, or paralysis. Hemorrhagic symptoms were defined as mucosal, gastrointestinal, or respiratory tract bleeding. Triggers of secondary HLH were determined based on physician's assessments. Laboratory parameters, including complete blood count, erythrocyte sedimentation rate, C-reactive protein, liver function test, lactate dehydrogenase, triglyceride, ferritin, fibrinogen, PTT, and d-dimer levels, were collected at the time of diagnosis and 1 week after diagnosis.

### Treatment outcomes

The primary outcome was the early mortality rate, defined as death that occurred within 30 days after diagnosis. Overall mortality was evaluated as death occurring during the follow-up period. Early treatment response was defined as the resolution of all clinical manifestations and normalization of HLH-related laboratory findings either complete blood count or other HLH-related laboratory parameters within 4 weeks (16).

### Statistical analysis

Descriptive statistics of demographic data and baseline laboratory parameters were expressed as a number with percentage and median with interquartile range (IQR). Comparisons between groups were performed using the chi-square or Fisher's exact test for categorical data and Mann–Whitney *U* test or Kruskal–Wallis test for continuous data. Predictors of treatment outcomes were analyzed using logistic regression, and the strength of predictors was described using the odds ratio (OR). Receiver operating characteristics (ROC) curve analysis was applied to select the optimal cut-off values of parameters. Associations between changes in laboratory parameters during 1 week after diagnosis and mortality were performed by mixed-model analysis. Survival was estimated by the Kaplan–Meier method with the log-rank test. A *p*-value <0.05 was considered statistically significant. The analysis was performed using IBM SPSS 24.0 statistical software.

## Results

Among 76 patients with HLH, 41 (54%) were females. The median age at diagnosis was 6.1 years (IQR 2–11.6). The most common HLH subtypes were IAHS, followed by MAS, primary HLH, and M-HLH (Table 1). The major causes of IAHS were viral infections with 45% Epstein–Barr virus (EBV), 24% dengue virus, and 13% cytomegalovirus (CMV). The common underlying rheumatic diseases in MAS were SJIA (67%) and SLE (33%). All cases of M-HLH occurred in patients with hematologic malignancies, mainly lymphoid malignancies (75%).

**Table 1:** Baseline clinical features and laboratory findings in HLH patients with different subtypes.

	**Primary HLH (*n* = 9)**	**IAHS (*n* = 38)**	**MAS (*n* = 21)**	**M-HLH (*n* = 8)**	***p*-value**
Age (median, IQR)	1.3 (0.6–3)	4.4 (1.7–10)	11.1 (6.7–11.8)	11.6 (1.5–14.8)	<0.001*
Male (*n*, %)	3 (33)	18 (47)	9 (43)	5 (63)	0.67
**Triggers/underlying diseases (** ***n*** **, %)**					
Epstein–Barr virus	N/A	17 (45)	N/A	N/A	
Dengue virus	N/A	9 (24)	N/A	N/A	
Cytomegalovirus	N/A	5 (13)	N/A	N/A	
Other pathogens	N/A	7 (18)	N/A	N/A	
SJIA	N/A	N/A	14 (67)	N/A	
SLE	N/A	N/A	7 (33)	N/A	
Lymphoid malignancy	N/A	N/A	N/A	6 (75)	
Myeloid malignancy	N/A	N/A	N/A	2 (25)	
**Clinical manifestations (** ***n*** **, %)**					
Fever	9 (100)	34 (90)	19 (91)	8 (100)	0.594
Hepatomegaly	9 (100)	36 (95)	16 (76)	6 (75)	0.063
Splenomegaly	8 (89)	30 (79)	10 (48)	6 (75)	0.047*
CNS involvement	5 (56)	10 (26)	6 (29)	0 (0)	0.083
Hemorrhages	7 (78)	13 (34)	4 (19)	1 (13)	0.011*
**Laboratory findings (median, IQR)**					
Hemoglobin (g/dL)	8.2 (7.2–9.8)	8.5 (7.8–9.8)	9.8 (8.9–10.9)	9.1 (7.4–10.6)	0.193
Leukocyte (/mm^3^)	4,000 (3,065–16,135)	2,480 (1,200–6,948)	4,000 (2,025–11,650)	2,800 (1,440–4,503)	0.087
Neutrophil (/mm^3^)	768 (288–2,804)	1,176 (213–2,790)	2,464 (1,447–6,490)	589 (21–1,903)	0.035*
Platelet (× 10^6^/mm^3^)	46 (25–61)	45 (23.75–87.25)	89 (46.5–150)	23 (16–218.25)	0.053
Fibrinogen (mg/dL)	86 (63–148)	142 (115–240)	218 (143–346)	206 (105–269)	0.014*
PTT (s), *n* = 72^a^	36.2 (30.6–74.9)	32.4 (28.4–40.9)	31.3 (26.9–38.2)	30.6 (26.7–37.6)	0.272
Aspartate transaminase (U/L)	549 (97–3,481)	227 (135–887)	138 (71–387)	149 (33–303)	0.24
Alanine transaminase (U/L)	317 (32–625)	118 (64–262)	108 (52–183)	78 (41–120)	0.297
Albumin (g/L)	25.6 (24.2–29.5)	25 (20.9–28.1)	27.5 (21.8–31.6)	27.1 (22.9–40.1)	0.423
Total bilirubin (mg/dL)	3.2 (0.7–12.7)	1.8 (1.1–4.5)	0.7 (0.3–1.8)	1.2 (0.6–6.7)	0.016*
Direct bilirubin (mg/dL)	2.6 (0.3–6.3)	1.3 (0.4–3.3)	0.4 (0.1–1.3)	0.4 (0.3–4.95)	0.05
Triglyceride (mg/dL)	357 (104–479)	249 (171–367)	228 (166–334)	187 (121–271)	0.52
Ferritin (ng/mL)	6,012 (810–46,079)	15,573 (5,443–40,000)	23,190 (6,628–40,000)	8,642 (1,382–15,119)	0.203

HLH, hemophagocytic lymphohistiocytosis; IAHS, infection-associated hemophagocytic lymphohistiocytosis; IQR, interquartile range; MAS, macrophage activation syndrome; M-HLH, malignancy-associated HLH; N, numbers; PTT, partial thromboplastin time; SJIA, systemic juvenile idiopathic arthritis; SLE, systemic lupus erythematosus. *p <0.05 was considered statistically significant. ^*a*^IAHS (n = 36), MAS (n = 20), M-HLH (n = 7).

Clinical characteristics and laboratory findings at the time of diagnosis were described in Table 1. Fever (92%) and hepatomegaly (88%) were commonly found in HLH patients. Patients with MAS had a lower prevalence of splenomegaly (48%) than patients with other HLH subtypes (*p* = 0.047). CNS involvement tended to be more prevalent (56%) in patients with primary HLH when compared to other HLH subtypes (*p* = 0.083). Among patients with clinical features of CNS involvement (*n* = 21), 5/6 patients who underwent CSF evaluation had pleocytosis or high protein level. Moreover, 10/14 patients who underwent brain imaging had abnormal results. In contrast, 14 patients without clinical features of CNS involvement had CSF evaluation or brain imaging which may be due to suspicious of infection or bleeding, and all patients had normal results. Patients with primary HLH had a higher prevalence of hemorrhage (78%) than patients with other subtypes (*p* = 0.011). The prevalence of hemorrhagic symptoms in MAS patients was 19%. SLE patients were more likely to have hemorrhagic symptoms (43%) compared with SJIA patients (7%). Laboratory findings showed that most HLH patients were thrombocytopenic (75%) and hyperferritinemic (93%) at the time of diagnosis. For primary HLH, patients had lower fibrinogen levels (*p* = 0.014) and higher total bilirubin levels (*p* = 0.016) than patients with other subtypes. Patients with MAS had significantly higher neutrophil counts (*p* = 0.035) and tended to have higher platelet counts (*p* = 0.053) compared with patients with other subtypes.

### Treatment and treatment outcomes

Twenty-five (33%) patients were treated according to the HLH 2004 protocol (26). Seven out of nine patients (78%) with primary HLH received the HLH 2004 protocol, and the remaining two died before receiving all medications in the protocol. Allogeneic hematopoietic stem cell transplantation was performed in two patients with primary HLH. Approximately 68% of IAHS patients required intravenous immunoglobulin (IVIG) combined with other immunosuppressive therapies, such as cyclosporine and etoposide, whereas 32% of patients were treated with IVIG monotherapy (Table 2). Patients with MAS were mainly treated with high-dose corticosteroids and cyclosporine with or without IVIG. Half of the patients with M-HLH received chemotherapies according to underlying malignancies, and the other half were treated with the HLH 2004 protocol.

**Table 2:** Treatment and treatment outcomes in HLH patients with different subtypes.

	**Primary HLH (*n* = 9)**	**IAHS (*n* = 38)**	**MAS (n = 21)**	**M-HLH (*n* = 8)**	***p*-value**
**Treatment (*****n**,* **%)**					
Pulse IVMP monotherapy	0 (0)	0 (0)	3 (14)	0 (0)	0.042*
IVIG monotherapy	0 (0)	12 (32)	0 (0)	0 (0)	0.005*
Combined therapy without etoposide	1 (11)	10 (26)	17 (81)	0 (0)	<0.001*
Etoposide-combined therapy	8 (89)	16 (42)	1 (5)	4 (50)	<0.001*
Chemotherapy^a^	0 (0)	0 (0)	0 (0)	4 (50)	<0.001*
**Treatment outcome (** ***n*** **, %)**					
Mortality	5 (56)	12 (32)	5 (24)	7 (88)	0.007*
Early mortality	3 (33)	5 (13)	4 (19)	1 (13)	0.502
Early treatment response	6 (67)	24 (63)	17 (81)	2 (25)	0.05

HLH, hemophagocytic lymphohistiocytosis; IAHS, infection-associated hemophagocytic lymphohistiocytosis; IVIG, intravenous immunoglobulin; IVMP, intravenous methylprednisolone, MAS, macrophage activation syndrome; M-HLH, malignancy-associated HLH. *p <0.05 was considered statistically significant. ^*a*^Chemotherapy according to underlying malignancies.

The overall mortality rate was 38% (29/76), with early death in 45% (13/29) (Table 2). The causes of death were infections (41%), uncontrolled bleeding (31%), and multiple organ failure (28%). M-HLH and primary HLH patients had high mortality rates of 88 and 56%, respectively. Among IAHS patients, the mortality rate of EBV and dengue-associated IAHS was 32, and 33%, respectively. In MAS patients, the overall mortality rate was 24%. SLE patients had a significantly higher mortality rate (57%) than SJIA patients (7%) (*p* = 0.025). For early mortality, there was no significant difference between HLH types. Patients with MAS tended to respond to treatment earlier than patients with other subtypes (early treatment response rate 81%, *p* = 0.05).

### Predictors of early mortality and mortality

We compared clinical features and laboratory findings between patients with and without early mortality, as shown in Supplementary Table 1. Then, we included the notable clinical manifestations and laboratory parameters classified using cut-off values from ROC curve analysis in the univariate analysis. The sensitivity, specificity and area under the ROC curve (AUC) values of the predictors in the regression model were shown in Supplementary Table 2. We found that CNS involvement, platelet counts <44 × 10^6^/mm^3^, PTT more than 35 s, and total bilirubin (TB) levels more than 1.8 mg/dL were predictors of early mortality. In the multivariate analysis, CNS involvement and platelet counts <44 × 10^6^/mm^3^ were independent predictors of early mortality with an OR of 13 [95% confidence interval (CI) 2–83, *p* = 0.007] and 8 [95%CI 1.3–49, *p* = 0.024], respectively (Table 3). Primary HLH was also included in the univariate regression analysis and the result showed OR 2.8 (95% CI 0.6–13.3), *p* = 0.183. We therefore did not include this factor in the multivariate regression analysis.

**Table 3:** Predictors of early mortality in HLH patients.

	**Univariate analysis**		**Multivariate analysis**	
	**OR (95%CI)**	***p*-value**	**OR (95%CI)**	***p*-value**
CNS involvement	15.8 (3.7–66.8)	<0.001*	13 (2–83)	0.007*
Platelet <44 × 10^6^/mm^3^	7.2 (1.8–28.9)	0.006*	8 (1.3–49)	0.024*
PTT > 35 s (*n* = 72)^a^	5.6 (1.4–22.6)	0.015*	2 (0.3–13.9)	0.48
Albumin <25 g/L	1.8 (0.5–5.9)	0.35		
Total bilirubin >1.8 mg/dL	11 (2.2–54.2)	0.003*	4.7 (0.7–32.7)	0.155

HLH, hemophagocytic lymphohistiocytosis; CNS, central nervous system; PTT, partial thromboplastin time; OR, odds ratio; CI, confidence interval. *p <0.05 was considered statistically significant. ^*a*^patients without early mortality (n = 59), patients with early mortality (n = 13).

**Figure 1:**
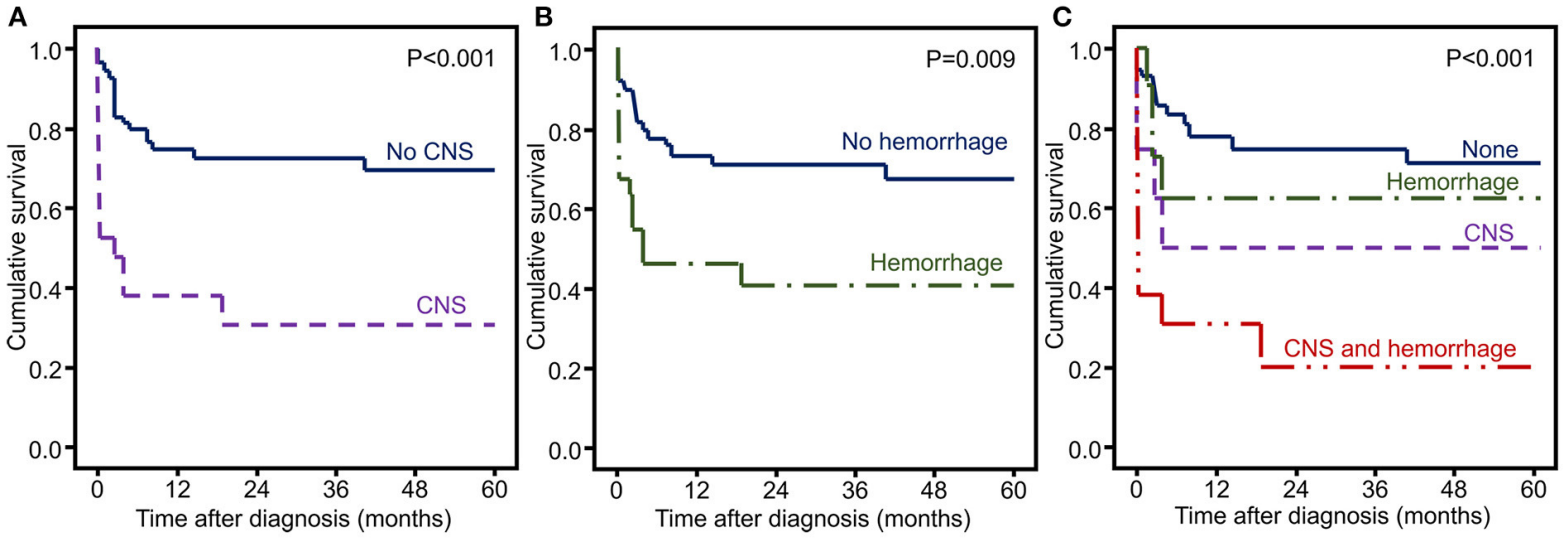
Kaplan–Meier curves of HLH patients with significant risk factors of mortality. **(A)** CNS involvement, **(B)** hemorrhagic symptoms, and **(C)** CNS and hemorrhagic symptoms.

The frequencies of CNS involvement and hemorrhagic symptoms were significantly higher in non-survivors than in survivors (48 vs. 15%, *p* = 0.002 and 48 vs. 23%, *p* = 0.025, respectively). Therefore, we included these two factors in the survival analysis. Overall survival was decreased in patients with CNS involvement (*p* < 0.001) and hemorrhagic symptoms (*p* = 0.009) (Figure 1).

### Predictors of early treatment response

Predictors related to early treatment response in the univariate analysis were no CNS involvement, platelet counts more than 44 × 10^6^/mm^3^, PTT <35 s, and TB levels <1.8 mg/dL. Subsequent multivariate analysis was performed, and the remaining predictors were no CNS involvement, platelet counts more than 44 × 10^6^/mm^3^, and TB levels <1.8 mg/dL (Table 4).

**Table 4:** Predictors of early treatment response in HLH patients.

	**Univariate analysis**		**Multivariate analysis**	
	**OR (95%CI)**	***p*-value**	**OR (95%CI)**	***p*-value**
No CNS involvement	6.5 (2.2–19.4)	0.001*	6.6 (1.5–28.8)	0.011*
Platelet ≥ 44 × 10^6^/mm^3^	6.2 (2.2–17.3)	0.001*	8 (2.1–30.9)	0.003*
PTT ≤ 35 s^a^	3 (1.1–8.1)	0.031*	1.6 (0.4–6.6)	0.481
Total bilirubin ≤ 1.8 mg/d	6.6 (2.3–18.6)	<0.001*	4 (1.1–14.8)	0.036*

HLH, hemophagocytic lymphohistiocytosis; CNS, central nervous system; PTT, partial thromboplastin time; OR, odds ratio; CI, confidence interval. *p <0.05 was considered statistically significant. ^*a*^patients with early treatment response (n = 46), patients without early treatment response (n = 26).

### Associations between changes in laboratory parameters and mortality

**Figure 2:**
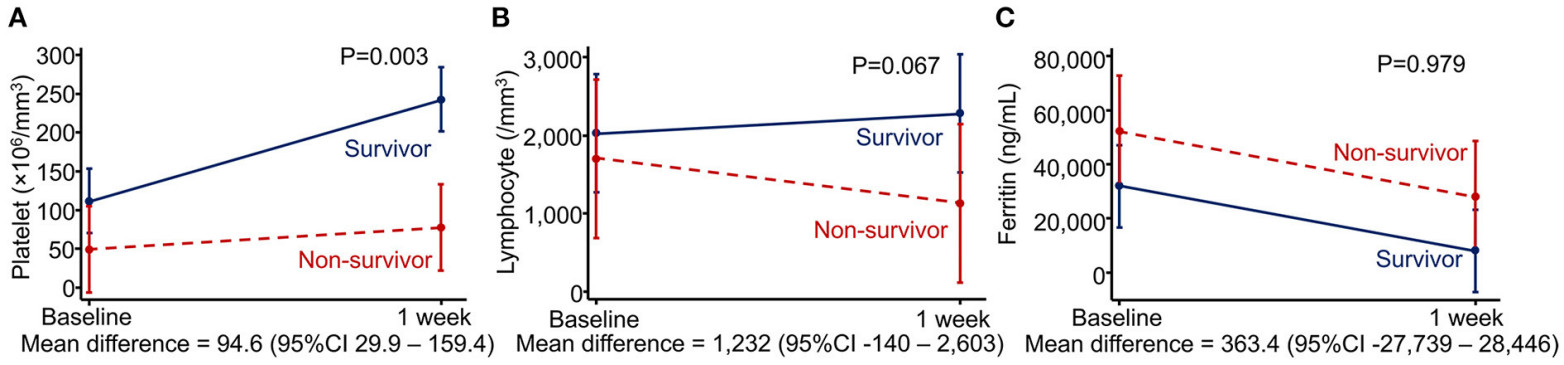
Associations between changes in laboratory parameters and mortality using mixed-model analysis. **(A)** Platelet count, **(B)** lymphocyte count, and **(C)** ferritin level.

We evaluated associations between changes in laboratory parameters during 1 week after diagnosis and mortality using mixed-model analysis. Non-survivors exhibited a significantly less improvement in platelet counts after the first week [mean increase 25.1 × 10^6^/mm^3^ (standard deviation 53.3)] than survivors (mean increase 119.7 × 10^6^/mm^3^ [standard deviation 169.4]) despite requiring more platelet transfusions during the first week than survivors [median (IQR) unit/kg, 1 (0.6–1.8) vs. 0 (0–0.4), *p* < 0.001]. The mean difference in platelet changes between the two groups was 94.6 × 10^6^/mm^3^ (95% CI 29.9–159.4, *p* = 0.003). Lymphocyte counts decreased in non-survivors but slightly increased in survivors. However, the difference was not statistically significant. Interestingly, ferritin levels decreased in both survivor and non-survivor groups, and the changes were not different between the two groups (Figure 2).

## Discussion

Our study demonstrated that mortality in HLH depends on the underlying disease that triggered this condition, particularly malignancies. Additionally, we revealed the predictive roles of mortality in HLH represented by CNS involvement (including seizures, irritability, altered consciousness, and paralysis) and changes in platelet counts. The predictors of early mortality were CNS involvement and platelet counts <44 × 10^6^/mm^3^, and the independent predictors of early treatment response were no CNS involvement, platelet counts more than 44 × 10^6^/mm^3^, and TB levels <1.8 mg/dL.

Consistent with previous reports, the two most common HLH types in this study were IAHS and MAS (16, 26). The most common pathogens triggering IAHS in previous studies were EBV and CMV (27, 28), whereas EBV followed by dengue virus were the most common pathogens triggering IAHS in our study. This might be because Thailand is in the endemic area of dengue infection. This was confirmed by the fact that most dengue-associated HLH cases were reported in South-East Asia (29, 30). The most frequent underlying rheumatic diseases in MAS were SJIA, followed by SLE. Patients with MAS had higher neutrophil counts and tended to have higher platelet counts. One potential explanation is that most MAS patients in this study were SJIA patients with higher baseline neutrophil and platelet counts than patients with other diseases. All MAS patients received high-dose corticosteroids, and more than 50% of patients required additional IVIG or cyclosporine, which was in line with previous studies (9, 31). Regarding M-HLH patients, most simultaneously had malignancies, predominately lymphoid malignancies, and 50–60% of patients were treated with the HLH protocol as first-line therapy (14, 27, 32).

The overall mortality rate in this study was 38%, and nearly half of deaths occurred within 30 days. This was similar to the 20–40% mortality rate and the majority of deaths occurring within 30 days in previous studies (1, 16, 22, 28). M-HLH had the highest mortality rate among all HLH types. Our 88% mortality rate of M-HLH was higher than the 56% mortality rate in the previous study of pediatric M-HLH (14). This might be because of the longer time to diagnosis and lower initial platelet counts in our patients than in patients from the previous study. The highest mortality among HLH subtypes was supported by numbers from previous studies in both children and adults (14, 27, 32–35). Primary HLH had the second highest mortality among HLH subtypes, despite the advanced treatment protocol. The mortality rate was approximately similar to that reported in a previous study (1). Interestingly, the mortality rate of dengue-associated HLH in our study (33%) was higher than the mortality rate of 14.6% in a recent meta-analysis (29). All dengue-associated non-survivors in our study had highly elevated AST and ALT, which were previously reported as risk factors of mortality (30). The other patients with dengue-associated HLH had milder disease courses and required only IVIG monotherapy. For MAS patients, those with SLE had a higher mortality rate than SJIA patients, potentially because of more hemorrhagic symptoms. Additionally, the hemorrhagic symptoms in our SLE patients were pulmonary hemorrhage symptoms, which are associated with high mortality (36). The reason for a higher incidence of hemorrhage may be lower platelet counts in patients with SLE than in patients with SJIA, although there was no statistical difference. The mortality rate reported in SLE patients with MAS was 5–16% (10, 37), which was lower than in our study. These differences might be because of lower platelet counts and higher rates of hemorrhagic symptoms in our SLE patients. The mortality rate of MAS in SJIA patients in our study was consistent with that reported in previous studies (9, 38, 39).

CNS involvement has been reported in 10–73% of HLH patients depending on the definition (15, 40, 41), which was similar to our study. Kim et al. found that positive neurological symptoms, elevated leukocytes in cerebrospinal fluid, and brain imaging abnormalities were associated with inferior survival compared with no CNS involvement (15). In addition, Zhao et al. (41) found that among HLH patients with CNS involvement, the presence of neurological symptoms, including seizures, irritability, somnolence, and unconsciousness, was the strongest prognostic factor for mortality compared with abnormal cerebrospinal fluid or abnormal brain imaging. Our study also showed that CNS involvement was associated with increased mortality rates in the survival analysis and was a predictor of early mortality. Moreover, absence of CNS involvement was a predictor of early treatment response. Although this study could not show statistical difference across the different HLH subtypes due to limited number of patients, patients with primary HLH had the highest proportion of CNS involvement. Also, more than half of those patients died within 30 days. From a neuropathologic study in children with HLH, the pathology in CNS can be staged based on the distribution pattern of lymphocytes and histiocytes or macrophage infiltration, finally leading to tissue necrosis. The more pronounced clinical symptoms were associated with more advanced stages of pathology (42). Therefore, CNS symptoms might reflect the severity of the disease and predict treatment outcomes.

Hemorrhagic symptoms were also associated with increased mortality in our study. The platelet count was one of the laboratory parameters associated with the risk of bleeding. Severe thrombocytopenia with a cut-off ranging from 30 to 75 × 10^6^/mm^3^ was associated with early mortality and poor treatment outcomes in children with HLH (16, 18, 19, 43). Similarly, our results found that a platelet count <44 × 10^6^/mm^3^ was another predictor of early death, and a platelet count more than this cut-off was a predictor of early treatment response. Thrombocytopenia in HLH results from hemophagocytosis and cytokine-induced bone marrow suppression, accompanied by excessive platelet destruction due to disseminated intravascular coagulation and hypersplenism (44). It reflects the severity of the condition (1, 44, 45).

Our study also found that patients with early mortality had higher levels of AST, ALT, TB, and direct bilirubin compared with patients without early mortality. In the ROC curve analysis, AST and ALT required very high cut-off values (>1,000 U/L) to increase the risk of early mortality; therefore, it might be impractical to use these parameters to identify patients at risk. Given that hyperbilirubinemia was previously reported as a potential predictor of treatment outcomes (17, 18, 22), we included TB levels in the logistic regression. TB levels <1.8 mg/dL were an independent predictor of early treatment response. All of these parameters, including PTT, were related to hepatic dysfunction; therefore, hepatic dysfunction might be more frequent in patients with poor treatment outcomes. Cytokines from macrophages, including IL-2, IFNγ, and TNF, cause inflammation in the liver, leading to sinusoidal dilatation, congestion, and elevated liver enzymes. In addition, lymphohistiocytic infiltration causes bile duct injury and cholestasis (46, 47). Hepatic injury in HLH results from excessive immune activation; therefore, its severity may affect treatment outcomes.

Previous studies reported that hyperferritinemia was associated with poor outcomes in both children and adults with HLH (19, 33, 43, 48, 49). Ferritin production is a compensatory mechanism that sequesters free Fe^2+^, which is released during red blood cell degradation in cytokine-mediated hemophagocytic processes. Thus, hyperferritinemia reflects the severity of cytokine storms (50). The cut-off values of ferritin ranged from more than 2,000 to 50,000 ng/mL (19, 33, 43, 48, 49). Baseline serum ferritin levels in this study were not statistically different between patients with and without early mortality; however, a significantly higher percentage of patients with early mortality had <35% reduction in ferritin levels over 1 week. Therefore, serial ferritin measurements during the disease course may provide a greater clinical benefit than the baseline measurement (51). Lin et al. (20) reported that ferritin decreases by <50% during 10 weeks after diagnosis were associated with an increased risk of mortality.

Our study also assessed changes in parameters from routine and basic laboratory data, such as hemoglobin, lymphocyte counts, and platelet counts, that can be used to identify high-risk patients. The increase in platelet counts during 1 week after diagnosis in non-survivors was less than that in survivors, despite more platelet transfusions. Refractory thrombocytopenia is likely owing to the vigorous consumption of platelets. This not only reflected the severity of HLH but also the increased risk of bleeding, leading to worse outcomes. Lymphocyte counts in the non-survivor group tended to decrease but were slightly increased in the survivor group. This might be explained by the fact that patients with lymphopenia were at a higher risk of infection, which was one of the causes of death in HLH.

The strength of this study is that our hospital is a tertiary care center that receives referrals from all over the country. We also have all types of specialists who see a variety of HLH patients. In addition, this study identified alterations in laboratory parameters during the disease course as predictive factors of treatment outcomes and early mortality. However, this study also has some limitations. First, genetic testing was not performed in patients with secondary HLH. Thus, we could not evaluate whether different mutations affect treatment outcome or not. Second, evaluation of CSF and brain imaging were lacking in most patients without clinical signs and symptoms of CNS involvement. We are aware that evaluation of CNS involvement which was mainly based on CNS signs and symptoms may not be adequate to identify patients with CNS involvement. Moreover, this study was performed in a single center and had a small sample size. Also, because of its retrospective design, some values were missing. Therefore, additional multi-center, prospective studies are recommended.

In conclusion, CNS involvement and low initial platelet counts are predictors of early mortality in children with HLH. Additionally, a slow rate of platelet increases during the first week after diagnosis was associated with mortality. Recognizing CNS symptoms at the initial presentation and serial monitoring of platelet counts during treatment courses may help identify the subgroups of HLH patients with a poor prognosis who need more intensive therapeutic modalities.

## Data availability statement

The raw data supporting the conclusions of this article will be made available by the authors, without undue reservation.

## Ethics statement

The studies involving human participants were reviewed and approved by the Ethics Committee of the Faculty of Medicine Ramathibodi Hospital, Mahidol University, Thailand (MURA2020/393). Written informed consent from the participants' legal guardian/next of kin was not required to participate in this study in accordance with the national legislation and the institutional requirements.

## Author contributions

SS contributed to the study conception and design. Material preparation, data collection, and analysis were performed by SH and SS. The first draft of the manuscript was written by SH and SS and all authors commented on previous versions of the manuscript. All authors read and approved the final manuscript.

## Funding

We receive funding from Faculty of Medicine Ramathibodi Hospital, Mahidol University for open access publication fees.

## Conflict of interest

The authors declare that the research was conducted in the absence of any commercial or financial relationships that could be construed as a potential conflict of interest.

## Publisher's note

All claims expressed in this article are solely those of the authors and do not necessarily represent those of their affiliated organizations, or those of the publisher, the editors and the reviewers. Any product that may be evaluated in this article, or claim that may be made by its manufacturer, is not guaranteed or endorsed by the publisher.

## Abbreviations

AST: aspartate aminotransferase
CMV: cytomegalovirus
CNS: central nervous system
CI: confidence interval
EBV: Ebstein–Barr virus
fHLH: familial hemophagocytic lymphohistiocytosis
HLH: hemophagocytic lymphohistiocytosis
IAHS: infection-associated hemophagocytic syndrome
IFN: interferon
IL: interleukin
IQR: interquartile range
IVIG: intravenous immunoglobulin
MAS: macrophage activation syndrome
M-HLH: malignancy-associated hemophagocytic syndrome
OR: odd ratio
PTT: partial thromboplastin time
ROC: receiver operating characteristics
SJIA: systemic juvenile idiopathic arthritis
SLE: systemic lupus erythematosus
TB: total bilirubin
TNF: tumor necrosis factor.
