# Supplementary material for: Central nervous system involvement and thrombocytopenia as predictors of mortality in children with hemophagocytic lymphohistiocytosis
Source: Front Pediatr. 2022 Sep 6;10:941318. doi: 10.3389/fped.2022.941318 (PMC9485874; doi:10.3389/fped.2022.941318)
Supplement: Supplementary file 1 [file Table_1.DOCX]

**Supplementary table**

**Table S1** Clinical features and laboratory findings between patients with and without early mortality

|  | **Early mortality**  (N=13) | **No early mortality**  (N=63) | ***p*-value** |
| --- | --- | --- | --- |
| CNS involvement (n, %) | 10 (77%) | 11 (18%) | <0.001* |
| Platelets (×10^6^/mm^3^) | 24 (18–41.5) | 64 (33–119) | 0.001* |
| PTT (s) (n=72)^a^ | 53.5 (35.8–74.9) | 30.9 (27.3–37.6) | <0.001* |
| Fibrinogen (mg/dL) | 129 (86–371) | 166 (117–244) | 0.629 |
| Total bilirubin (mg/dL) | 5.7 (2–11.75) | 1.2 (0.6–3) | 0.001* |
| Albumin (g/L) | 22.2 (19.6–27.2) | 26.3 (23.1–30.8) | 0.136 |
| Ferritin (ng/mL) | 29,394 (2,805–99,172) | 12,332 (5,133–39,056) | 0.452 |
| Ferritin change in 1 week (ng/mL)^b^ | 2,558 (-59,763–24,067) | -7,269 (-35,216–[-1,667]) | 0.177 |
| %Ferritin at 1 week compared with baseline (%) (n=56)^b^ | 128.3 (12.1–184.3) | 22.9 (9.8–62.2) | 0.088 |
| Ferritin decline <35%  in 1 week (n=56)^b^ | 6 (46%) | 11 (17%) | 0.017* |

CNS, central nervous system; PTT, partial thromboplastin time

**p* <0.05 was considered statistically significant

^a^Patients without early mortality (n=59), patients with early mortality (n=13)

^b^Patients without early mortality (n=47), patients with early mortality (n=9)
