# Supplementary material for: Central nervous system involvement and thrombocytopenia as predictors of mortality in children with hemophagocytic lymphohistiocytosis
Source: Front Pediatr. 2022 Sep 6;10:941318. doi: 10.3389/fped.2022.941318 (PMC9485874; doi:10.3389/fped.2022.941318)
Supplement: Supplementary file 2 [file Table_2.DOCX]

**TABLE S2** Sensitivity, specificity and area under the receiver operating characteristics (ROC) curve values of the indicators of early mortality

|  | **Sensitivity (%)** | **Specificity (%)** | **AUC value** |
| --- | --- | --- | --- |
| Platelet <44×10^6^/mm^3^ | 84.6 | 66.7 | 0.784 |
| PTT >35 s (n=72) | 76.9 | 66.1 | 0.819 |
| Total bilirubin >1.8 mg/dL | 84.6 | 66.7 | 0.796 |
| Albumin <25 g/L | 69.2 | 52.4 | 0.632 |

AUC, area under the ROC curve
